# Feeding *Cyprinus carpio *with infectious materials mediates cyprinid herpesvirus 3 entry through infection of pharyngeal periodontal mucosa

**DOI:** 10.1186/1297-9716-43-6

**Published:** 2012-01-25

**Authors:** Guillaume Fournier, Maxime Boutier, Victor Stalin Raj, Jan Mast, Eric Parmentier, Pierre Vanderwalle, Dominique Peeters, François Lieffrig, Frédéric Farnir, Laurent Gillet, Alain Vanderplasschen

**Affiliations:** 1 Immunology-Vaccinology (B43b), Department of Infectious and Parasitic Diseases (B43b), Faculty of Veterinary Medicine, University of Liège, 4000 Liège, Belgium; 2 Department Biocontrole, Research Unit Electron Microscopy, VAR-CODA-CERVA, Groeselenberg 99, B-1180 Ukkel, Belgium; 3 Laboratoire de Morphologie Fonctionnelle et Evolutive, B6c, University of Liège, B-4000 Liège, Belgium; 4 Médecine Interne des Animaux de Compagnie, B44, Faculty of Veterinary Medicine, University of Liège, B-4000 Liège, Belgium; 5 CERgroupe, rue du Carmel 1, B-6900 Marloie, Belgium; 6 Biostatistics (B43), Faculty of Veterinary Medicine, University of Liège, 4000 Liège, Belgium

## Abstract

Cyprinid herpesvirus 3 (CyHV-3), also known as Koi herpesvirus, is the etiological agent of a mortal disease in common and koi carp. Recently, we investigated the entry of CyHV-3 in carp using bioluminescence imaging and a CyHV-3 recombinant strain expressing luciferase (LUC). We demonstrated that the skin is the major portal of entry after inoculation of carp by immersion in water containing CyHV-3. While this model of infection mimics some natural conditions in which infection takes place, other epidemiological conditions could favour entry of virus through the digestive tract. Here, we investigated whether ingestion of infectious materials mediates CyHV-3 entry through the digestive tract. Carp were fed with materials contaminated with the CyHV-3 LUC recombinant (oral contamination) or immersed in water containing the virus (contamination by immersion). Bioluminescence imaging analyses performed at different times post-infection led to the following observations: (i) the pharyngeal periodontal mucosa is the major portal of entry after oral contamination, while the skin is the major portal of entry after contamination by immersion. (ii) Both modes of inoculation led to the spreading of the infection to the various organs tested. However, the timing and the sequence in which some of the organs turned positive were different between the two modes of inoculation. Finally, we compared the disease induced by the two inoculation modes. They led to comparable clinical signs and mortality rate. The results of the present study suggest that, based on epidemiological conditions, CyHV-3 can enter carp either by skin or periodontal pharyngeal mucosal infection.

## Introduction

The Cyprinid herpesvirus 3 (CyHV-3; species *Cyprinid herpesvirus 3*, genus *Cyprinivirus*, family *Alloherpesviridae*, order *Herpesvirales*), also known as koi herpesvirus, is the aetiological agent of a contagious and mortal disease in common (*Cyprinus carpio carpio*) and koi (*Cyprinus carpio koi*) carp [1-5]. Since its emergence, in the late 1990s, CyHV-3 has caused severe economic losses in both common and koi carp culture industries worldwide [4,6,7].

The recent publication of the CyHV-3 sequence [8], together with the cloning of its genome as an infectious bacterial artificial chromosome (BAC) [9], allowed the production of CyHV-3 recombinant strains. Recently, we took advantage of these advances to construct a luciferase (LUC)-expressing recombinant strain by intergenic insertion of a LUC expression cassette [10]. Using this recombinant strain, bioluminescent imaging, and an original system to perform percutaneous infection restricted to the posterior part of the fish, we showed that the skin covering the fins and the body, and not the gills, is the major portal of entry after inoculation by immersion in water containing the virus [10]. This study, together with an earlier report addressing the portal of entry of a rhabdovirus (infectious hematopoietic necrosis virus) in salmonids [11], suggests that the skin of teleost fish is an efficient portal of entry for certain viruses.

The skin is the major portal of entry of CyHV-3 in carp after inoculation by immersion in water containing the virus [10]. While this model of infection certainly mimics some natural condition of infections, other conditions could favor entry of the virus through the digestive tract. Firstly, droppings from infected carp have been shown to contain infectious virus [12]. Ingestion of infectious droppings or food contaminated by droppings by naïve subjects could represent a source of oral inoculation. Secondly, carp express cannibalistic and necrophagous behaviour. By ingestion of infectious tissues of CyHV-3 infected carp [10,13], naïve subjects could infect themselves through the oral route. Finally, recent studies performed in habitats with CyHV-3 history suggested that aquatic invertebrates feeding by water filtration could accumulate and store CyHV-3 [14]. Ingestion of contaminated invertebrates could represent another possible source of CyHV-3 oral infection. Together with the observation that CyHV-3 replicates intensively in the intestine during the disease it causes [12], the possible sources of CyHV-3 oral contamination listed above stimulated the study of the role of carp digestive tract as a possible portal of entry for the virus.

**Figure 1:**
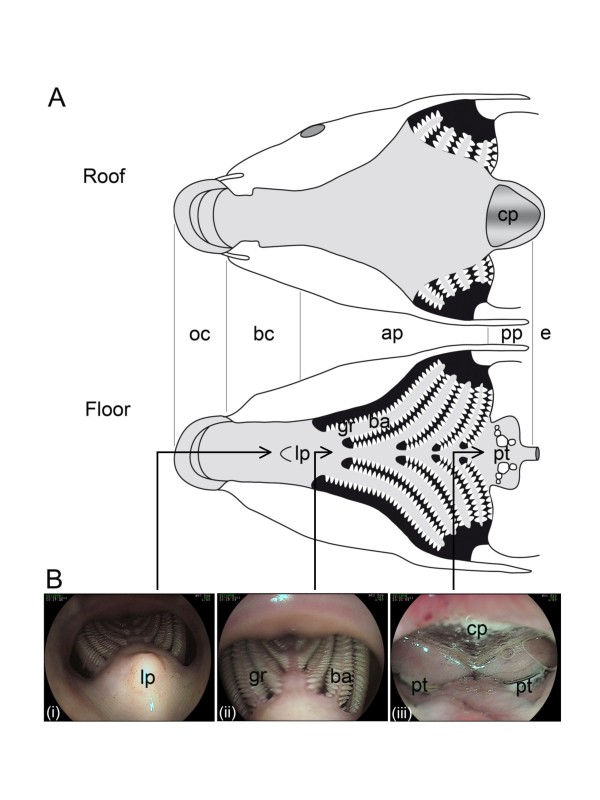
**Oropharyngeal cavity of carp**. (A) Schematic representation of the roof and floor of the carp oropharyngeal cavity (adapted from [17]). The cavity is subdivided into four sections: oral cavity (oc), buccal cavity (bc), anterior pharynx (ap), posterior pharynx (pp). esophagus (e); lp, lingual process; ba, branchial arch; gr, gill raker; pt, pharyngeal teeth; and cp, chewing pad. (B) Endoscopy views of carp oropharyngeal cavity. A 2 kg carp was anesthetized before exploration of its oropharyngeal cavity by endoscopy. Panels i-iii illustrate bc, ap, and pp, respectively.

The digestive tract of common carp is composed of the oropharyngeal cavity, the esophagus and the intestine [15-17]. The oropharyngeal cavity is subdivided into four sections: the oral cavity, the buccal cavity, the anterior pharynx (syn. branchial cavity) and the posterior pharynx (syn. chewing cavity) (Figure 1). The three first sections are involved in respiration and food selection, while the posterior pharynx between pharyngeal teeth and chewing pad is involved in mastication. Common carp are stomach-less fish. The short esophagus connects the posterior pharynx to the anterior part of the intestine also called pseudogaster [18].

In the present study, we investigated the role of the carp digestive tract as a viral portal of entry using bioluminescence imaging. We found that feeding carp with infectious materials induces CyHV-3 entry through infection of the pharyngeal periodontal mucosa. The results of the present study suggest that, based on epidemiological conditions, CyHV-3 can enter carp either by skin (immersion in infectious water) or periodontal pharyngeal mucosal infection (ingestion of infectious materials).

## Materials and methods

### Virus

The KHV FL BAC 136 LUC TK revertant strain of CyHV-3, hereafter called LUC strain, was described previously [10]. This recombinant strain encodes a firefly luciferase (LUC) expression cassette inserted in the intergenic region between open reading frame (ORF) 136 and ORF137.

### Fish

Common carp *(Cyprinus carpio carpio) *(CEFRA, University of Liège, Belgium) with an average weight of 10 g and 100 g were kept in 60-liter tanks at 24°C. Microbiological, parasitical and clinical examinations of the fish just before the experiments demonstrated that these fish were fully healthy. Two common carp with a weight of 2 and 3 kg were collected from a private pond.

### CyHV-3 inoculation of carp

Common carp were inoculated by one of two different inoculation modes. To mimic contamination through infectious water, 10 g fish (5 fish/L) were immersed for 2 h in water containing 300 PFU/mL of the CyHV-3 LUC strain. At the end of the contamination period, the fish were returned to the larger tank. To mimic contamination by the oral route, (10 g and 100 g) fish kept individually in 2 L of water were fed with three pellets of food (Ichi Food Summer mini 2-3 mm; Aquatic Science) contaminated with the CyHV-3 LUC strain. Food pellets were contaminated with CyHV-3 by immersion of ten food pellets per mL of CyHV-3 LUC strain (2.8 × 10^5 ^PFU/mL) for 10 min. Pellets were distributed to fish immediately after incubation. To determine the number of infectious particles contained in a pellet, 5 contaminated pellets (in triplicate) were disrupted by flushing through a 5 mL pipette in 5 mL of minimum essential medium (MEM) (Invitrogen, Merelbeke, Belgium). After centrifugation (6000 *g *for 20 min at 4°C), the supernatant was collected and sterilized by filtration through a 0.45 μm filter (0.45 μm filter PES, VWR). Infectious particles were then titrated as described elsewhere [10]. Titration of CyHV-3 in the pellets revealed that they contained 754.5 ± 59.6 PFU/pellet (mean ± SE of triplicate measurements). Fish were regrouped in the larger tank after ingestion of the food. The animal studies presented in this manuscript have been accredited by the local ethics committee of the University of Liège, Belgium (N° LA1610008/810, 1059 and 1063).

### Bioluminescence imaging

Imaging of firefly (*Photinus pyralis*) LUC was performed using an "in vivo imaging system" (IVIS) (IVIS^®^spectrum, Xenogen, Caliper LifeSciences, Hopkinton, Massachusetts, USA) as described previously [10]. Fish were anesthetized with benzocaine (50 mg/L of water). Fifteen minutes before bioluminescence analysis, D-luciferin (150 mg/kg body weight) (Xenogen, Caliper LifeSciences, Hopkinton, Massachusetts, USA) was administered by intraperitoneal injection. Each fish was analysed in vivo lying on its right and its left side and ex vivo after euthanasia and dissection. All the images presented in this study were acquired using a field view of 15 cm, an auto-exposure time with a maximum of 1 minute, a binning factor of 4 and a f/stop of 1. Relative intensities of transmitted light from bioluminescence were represented as a pseudocolor image ranging from violet (least intense) to red (most intense). Corresponding grey-scale photographs and color luciferase images were superimposed using the LivingImage analysis software (Xenogen, Caliper LifeSciences, Hopkinton, Massachusetts, USA). For quantitative comparisons, the Living Image software (Caliper Life Sciences) was used to obtain the total flux (p.s^-1^) over each region of interest (ROI). All the ROI automatically identified by the IVIS software as positive (Figure 4a) were standing out against background with a difference of at least 3 log.

### Transmission electron microscopy

Samples were fixed in 0.1% glutaraldehyde (Sigma-Aldrich, Saint Louis, Missouri, USA). Epon blocks and sections were prepared as described elsewhere for histological and electron microscopic examination [19]. Sections were analysed using a Tecnai Spirit transmission electron microscope (FEI, Eindhoven, The Netherlands), and electron micrographs were taken using a bottom-mounted 4-by-4 K Eagle camera (FEI).

### Statistical analyses

A possible difference in the dynamics of IVIS positive organs (Figure 4b) or in the dynamics of mortality (Figure 5) was tested using a permutation test as follows. Firstly, occurrences were recorded in the real dataset. Then, in successive repetitions (1000 or 10 000) of the same procedure, these occurrences were randomly allocated to each of the 2 groups (immersion and oral inoculation), so mimicking the observed data but without introducing any systematic difference between the 2 groups. A measure of the global difference between the 2 curves - taken as the sum over the days of the absolute difference at any given day - was then obtained for these shuffled dataset and compared to the really observed one. The proportion of shuffled datasets with a measure greater or equal to the real difference was then taken as the *p*-value.

## Results and Discussion

### CyHV-3 portal of entry after inoculation by immersion in infectious water or by feeding with contaminated materials

**Figure 2:**
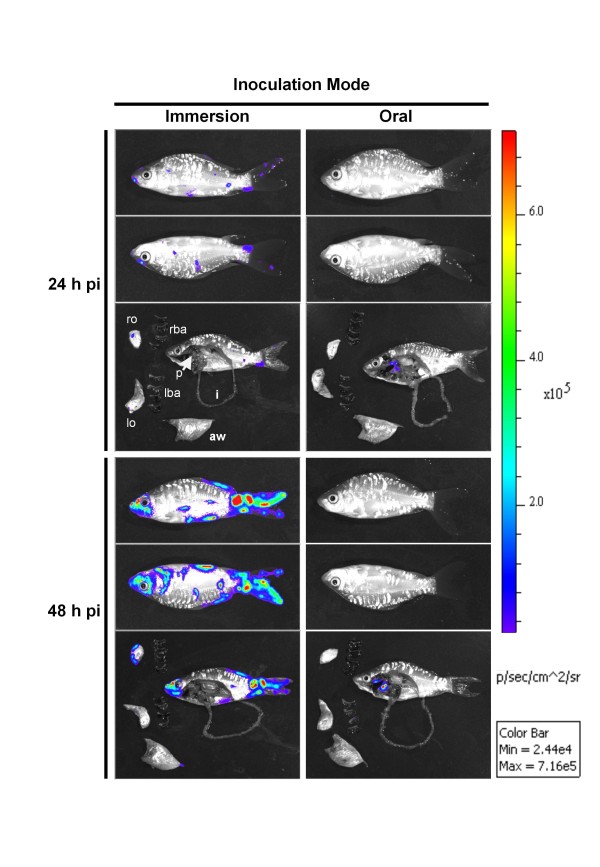
**The portal of entry of CyHV-3 in carp analysed by bioluminescence imaging**. Two groups of fish (mean weight 10 g) were infected with the CyHV-3 LUC strain either by bathing them in water containing the virus (Immersion, left column) or by feeding them with food pellets contaminated with the virus (Oral, right column). At the indicated time pi, six fish per group were analysed by bioluminescence IVIS. Each fish was analysed lying on its right and its left side. To analyze internal signals, fish were euthanized and dissected immediately after in vivo bioluminescence imaging. Dissected fishes and isolated organs were analysed for ex vivo bioluminescence. The analysis of one fish is presented for each time point and inoculation mode. Pictures collected over the course of this experiment are presented with a standardized minimum and maximum threshold value for photon flux. rba, right branchial arches; lba, left branchial arches; ro, right operculum; lo, left operculum; p, pharynx; aw, abdominal wall; i, intestine.

**Figure 3:**
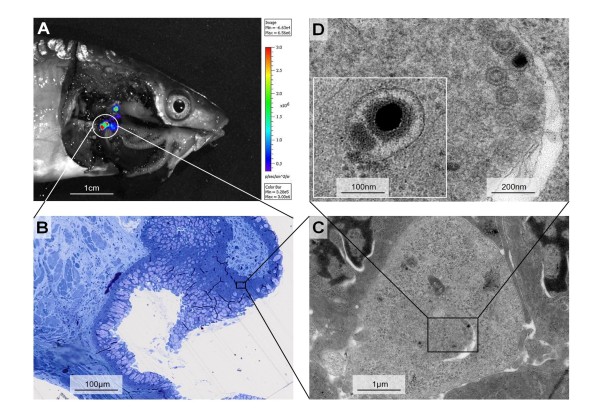
**In situ localization of LUC activity and detection of viral replication in carp periodontal pharyngeal mucosa**. Carp weighing 100 g were fed with food pellets contaminated with the CyHV-3 LUC strain. At 2 dpi, carp were anesthetized, injected with luciferine, and euthanized immediately before dissection of the oropharyngeal cavity. Dissected fish were analysed for ex vivo bioluminescence (A). A fragment of periodontal pharyngeal mucosa emitting bioluminescence was harvested and processed for histological (B) and electron microscopy analysis (C and D). Panel C shows low magnification of the epithelium. Panel D shows one representative infected epithelial cell at higher magnification.

In the present study, we investigated the role of carp digestive tract as a putative portal of entry for CyHV-3 using bioluminescence imaging. Carp were infected with the CyHV-3 LUC strain using two modes of inoculation: immersion in water containing the virus or feeding with contaminated materials (Figure 2). Fish were analysed by IVIS 24 and 48 h post-infection. Because photon emission is drastically attenuated in fish tissues [10], each fish was analysed in vivo lying on its right and then left side, and ex vivo after euthanasia and dissection. The results can be summarized as follows: in fish inoculated by immersion, 5 out of 6 fish analysed 1 day post-infection (dpi) expressed at least one focal source of light on the body surface. The signals were detected from various anatomic sites of the fish body, but principally on the fins. Analyses performed 2 dpi revealed that all of the fish had LUC signals on their surface (*n *= 6). In comparison to day 1, the number and the intensity of light focal sources detected 2 dpi increased in number and intensity. None of the fish inoculated by immersion expressed internal LUC signals neither at 1 dpi nor at 2 dpi. These observations confirmed our former results [10] demonstrating that the skin is the major portal of entry of CyHV-3 after inoculation by immersion in infectious water. Analysis of fish inoculated by ingestion of infectious materials led to unexpected results (Figure 2, oral inoculation mode). While none of the six fish analysed 1 dpi displayed LUC signals on the skin, one of them expressed LUC at the posterior part of the carp pharyngeal cavity. At 2 dpi, all of the analysed fish (*n *= 6) had intense light-emitting foci in the posterior part of the pharyngeal cavity. For 5 of the fish, no other LUC signal was detected elsewhere on or in the body (Figure 2). In addition to a strong pharyngeal signal, one single fish expressed a focal source of light on one branchial arch (data not shown). Because of the small size of the common carp used for this experiment, it was difficult to identify precisely the site of light emission within the pharyngeal cavity. Consequently this part of the experiment was repeated with larger carp (100 g, *n *= 5) (Figure 3).

**Figure 4:**
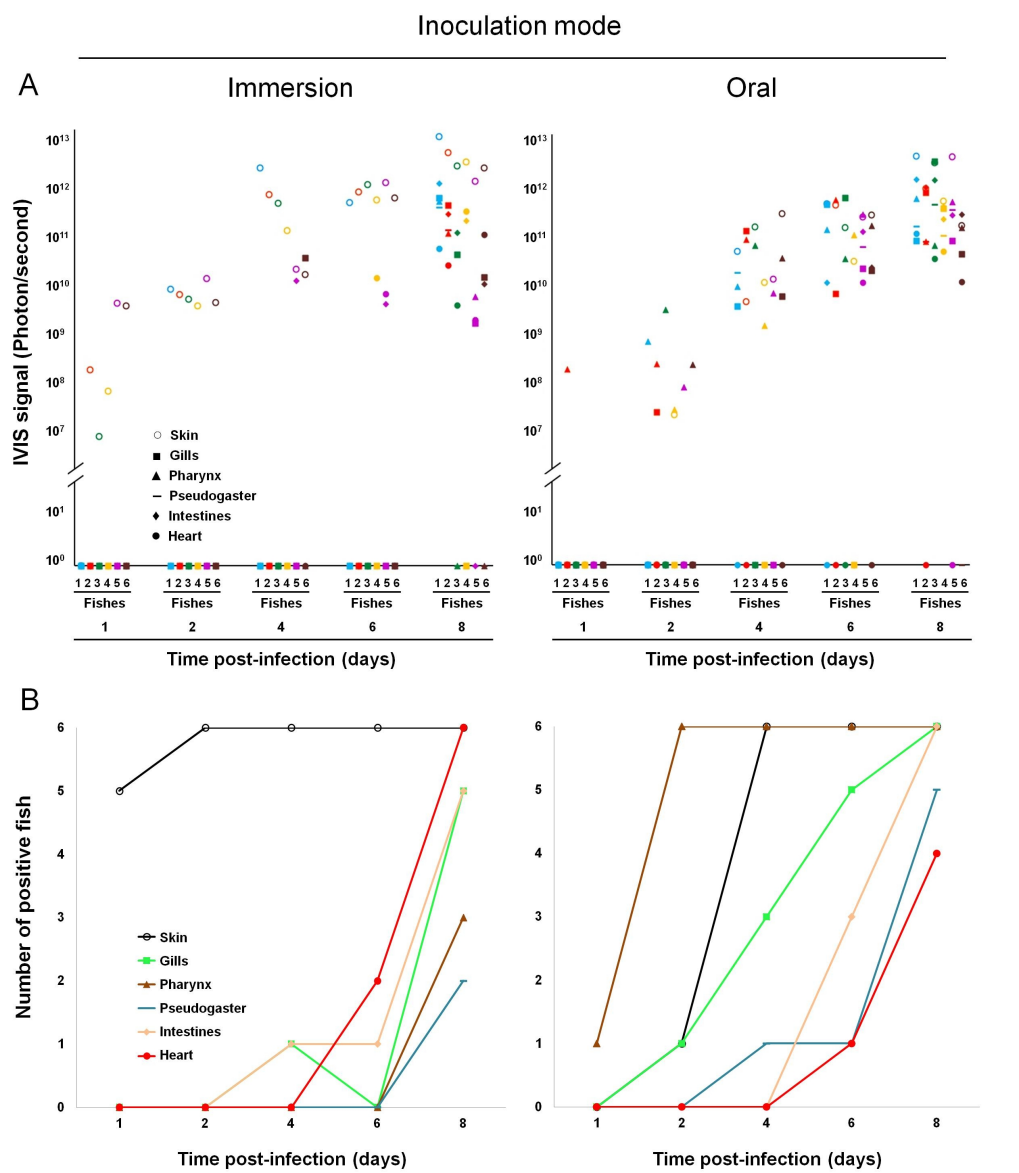
**Progression of CyHV-3 infection in carp analysed by bioluminescence imaging**. Two groups of fish (mean weight of 10 g) were infected with the CyHV-3 LUC strain either by bathing them in water containing the virus (Immersion, left column) or by feeding them with food pellets contaminated with the virus (Oral, right column). At the indicated time post-infection, six fish per group were analysed by in vivo and ex vivo bioluminescence imaging. A/For each fish, the IVIS signal (Photon/second) was determined for several organs (skin, gills, pharynx, pseudogaster, intestine, and heart) as described in the materials and methods. B/For each analysed organ, the number of positive fish is presented according to time post-infection. This experiment is representative of two independent experiments.

**Figure 5:**
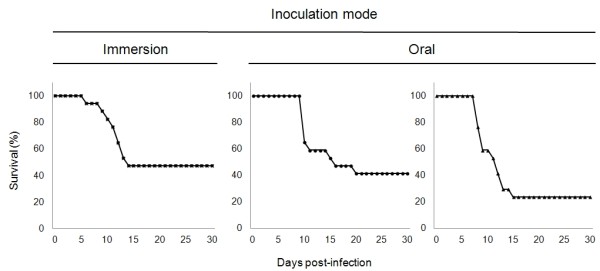
**Survival rates of carp infected with the CyHV-3 LUC strain**. On day zero, three groups of fish, each consisting of 17 common carp (mean weight of 10 g) kept in separate tanks, were infected either by bathing them in water containing the virus (left graph, immersion) or by feeding them with food pellets contaminated with the virus (middle and right graphs, Oral) as described in Materials and Methods. The fish were examined daily for clinical signs of CyHV-3 disease, and dead fish were removed. The percentage of survival is expressed according to time post-infection.

The results obtained were consistent with those generated in smaller fish. All fish (*n *= 5) that we analysed 2 dpi expressed LUC at the posterior part of the pharyngeal cavity. Ex vivo bioluminescent analysis of dissected pharyngeal cavities revealed that luciferase expression was localized to the protruding periodontal pharyngeal mucosa between the pharyngeal teeth and the chewing pad (Figure 3a). This mucosa forms protruding foliaceous papillae within the pharyngeal cavity (Figure 1b, panel iii) [17]. The stratified oropharyngeal epithelium consists of common epithelial cells, as well as several specialized cells, such as mucous cells, club cells, chloride cells, and sensory cells [17]. Next, to investigate whether LUC expression detected on the pharyngeal mucosa was associated with viral replication, and if so to identify the cell type(s) supporting the infection, a biopsy specimen of positive mucosa was analysed by electron microscopy (Figure 3c-d). A detailed examination of ultrathin sections revealed cells supporting viral replication in the mucosa epithelium. The infected cells could be identified at low magnification based on their less-electron-dense cytoplasm and nucleus. Viral capsids and enveloped particles were observed in the nuclei and the cytosol of the infected cells, respectively. All of the infected cells that we detected were common epithelial cells.

While common epithelial cells are abundant throughout the oropharyngeal cavity, LUC signal was restricted to the pharyngeal periodontal mucosa (Figures 2 and 3). There are two hypotheses that could explain this observation. First, the common epithelial cells in this area could express cell-surface molecules that make them highly sensitive to CyHV-3 infection. A second, more likely, hypothesis relies on a mechanical phenomenon: during mastication, mucus removal and/or microlesions could be induced in protruding foliaceous papillae by food and/or the pharyngeal teeth, creating an efficient portal of entry for CyHV-3. This hypothesis is consistent with our recent observation that skin mucus removal with or without associated epidermal lesions drastically enhance CyHV-3 entry [20]. To investigate the likelihood of this hypothesis, we recorded carp mastication movements by video endoscopy (additional file Video 1, second section). The video showed that the protruding periodontal mucosa covers pharyngeal teeth and is likely to be affected by mastication.

CyHV-3 replicates intensively in the intestine and is excreted in droppings during the disease it causes [12] (see data of Figure 4 below). However, the data of the present study suggest that the intestine does not act as a portal of entry for CyHV-3 after oral contamination. Several hypotheses could explain these observations. Firstly, it is possible that CyHV-3 is quickly inactivated in the lumen of the anterior part of the digestive tract. Intestinal mucus and/or secreted enzymes could inactivate CyHV-3 infectivity. This hypothesis is supported by the recent observation that epidermal soluble mucus extract is able to neutralise CyHV-3 infectivity [20]. Secondly, it is possible that enterocytes which are polarized cells express receptor(s) for CyHV-3 entry on their basolateral plasma membrane but not on their luminal apical membrane.

### CyHV-3 pathogenesis after inoculation by immersion in infectious water or by feeding with contaminated materials

The results presented above suggest that according to epidemiological conditions, CyHV-3 enters carp through skin (immersion in infectious water) or periodontal pharyngeal infection (feeding on contaminated materials). In the second part of this study, we investigated whether the two modes of inoculation induce similar CyHV-3 disease. First, we investigated by bioluminescence imaging how the virus spreads from the portal of entry to secondary sites of replications. Two groups of fish were infected either by immersion in infectious water or by feeding with infectious materials (Figure 4). At 1, 2, 4, 6 and 8 dpi, 6 fish per group were analysed by bioluminescence imaging and the emission of photons was quantified for selected tissues/organs (Figure 4a). Figure 4b illustrates the dynamics of the infection within tested organs according to the two modes of inoculation. Statistical analyses of these data (permutation test, 10 000 permutations) demonstrated that the dynamic of positive organs differed significantly between the two modes of inoculation for the skin (*p *= 0.004) and the pharynx (*p *= 0.0025), while gills (*p *= 0.059) almost reached significance. Pseudogaster (*p *= 0.237), guts (*p *= 0.832) and heart (*p *= 0.833) were found not to differ.

The analyses performed at 1 and 2 dpi confirmed that the skin and the periodontal pharyngeal mucosa are the major portal of entry after inoculation by immersion in infectious water and by feeding with infectious materials, respectively. In the latter case, soon after positivity of the pharynx, the skin became positive closely followed by the gills (Figure 4b). Two hypotheses could explain that the skin is the second place the virus is seen after the pharynx following oral exposure. Firstly, it is possible that the skin signal detected 2 dpi represents a low level of infection that occurred at the time of feeding. Indeed, it is likely that contaminated food pellets released virions in the water before they were ingested by carp. Due to the low concentration of the virus in the water, the resulting skin infection was perhaps not detected 1 dpi but rather at 2 dpi. Secondly, it is possible that the skin signal observed on day 2 pi represents spreading of the viral infection on the fish surface from the periodontal pharyngeal mucosa.

In the fish inoculated by immersion, the gills were not positive until day 6 when they were positive earlier in the oral route (Figure 4). The most likely explanation of this observation is that the earlier infection of the gills observed in the oral route represents the spreading of the viral infection from the pharyngeal mucosa by continuity of tissue; while the infection of the gills observed after bath exposure could reflect the systemic spreading of the infection.

Next, we investigated whether the mode of inoculation (immersion versus oral) could affect the disease induced in term of clinical signs and mortality rate (Figure 5). Three groups of fish each consisting of 17 carp were inoculated either by immersion in infectious water (1 group) or by feeding with contaminated materials (2 groups). Daily examination of carp did not reveal any significant difference between the two modes of infection. All groups of fish expressed the clinical signs associated with CyHV-3 disease, including apathy, folding of the dorsal fin, increased mucus secretion, suffocation, erratic swimming, and loss of equilibrium. The intensities of the clinical signs were comparable in the three groups. Comparison of the survival rates between the three groups led to the following conclusions. Survival curves for the two orally inoculated samples were analysed using a binomial comparison of the survival rates 30 days after inoculation. No significant difference was found (*p *= 0.08). Accordingly, the two samples were pooled and the survival rate after 30 days was compared between the pooled sample and the sample with inoculation by immersion. Again, a binomial test confirmed that no significant survival rate difference can be detected (*p *= 0.15). A possible difference in the mortality dynamics according to the mode of inoculation was then tested using a permutation test (1000 permutations). The obtained p-value of *p *= 0.331 showed that no significant difference exists between the two inoculation mode dynamics.

The results presented above suggest that CyHV-3 induces a comparable disease after entry through infection of the skin or periodontal pharyngeal mucosa (Figures 4 and 5). Based on the IVIS data of the present study and earlier studies [12,21], we propose a model for CyHV-3 pathogenesis. According to epidemiological conditions (immersion in water containing the virus or ingestion of infectious materials), CyHV-3 enters fish through skin or pharyngeal periodontal infection (Figure 2) [10,20]. Earlier reports based on PCR analysis described an early and fast systemic spread of the virus in infected fish [13,22-24], while our IVIS data suggested that active replication within secondary sites occurs only 4-6 days after contamination. To explain these data we propose that the rapid (2 dpi) and systemic dissemination observed by PCR reflects the secondary infection of blood cells [24], which could not be detected by bioluminescence imaging. Infected blood cells supporting a replicative infection could act as Trojan horse for the virus leading to a systemic distribution of the virus within the infected host. Associated with this phase of systemic distribution, the virus could reach secondary sites of replication among which some will contribute to excretion of infectious particles in the environment (intestine and gills). A recent study on CyHV-3 pathogenesis support the role of infected blood cells described above both during clinical infection as well as during latency [21].

In conclusion, this study demonstrated that according to epidemiological conditions, CyHV-3 can enter carp either through infection of the skin (immersion in infectious water) or through infection of the pharyngeal periodontal mucosa (feeding on infectious materials). The existence of these two portal of entry adapted to different epidemiological conditions most probably contributes to the high contagious nature of the virus.

## List of Abbreviations

Ap: anterior pharynx; Aw: abdominal wall; Ba: branchial arch; Bc: buccal cavity; Cp: chewing pad; CyHV-3: cyprinid herpesvirus-3; Dpi: day post-infection; E: esophagus; Gr: gill raker; I: intestine; IVIS: in vivo imaging system; Lba: left branchial arches; Lo: left operculum; Lp: lingual process; LUC: luciferase; Oc: oral cavity; ORF: open reading frame; P: pharynx; PFU: plaque forming unit; Pp: posterior pharynx; Pt: pharyngeal teeth; Rba: right branchial arches; Ro: right operculum; ROI: region of interest.

## Competing interests

The authors declare that they have no competing interests.

## Authors' contributions

GF, MB and VSR contributed to the design of the study. GF, MB, VSR performed the experiments and drafted the figures. EP and PV provided expertise in the oropharyngeal cavity of cyprinids. DP performed endoscopy exploration of carp pharyngeal cavity. FL controlled the sanitary statue of the carp and took care of zootechnique aspects. JM performed electron microscopy analyses. FF performed statistical analyses. AV conceived the study and drafted the manuscript. All authors read and approved the final manuscript.

## Supplementary Material

Additional file 1 **Video 1: Exploration of carp oropharyngeal cavity by endoscopy**. First section: endoscopy of buccal and pharyngeal cavities of *C. carpio*. A 2 kg common carp was anesthetized to allow endoscopic exploration of oropharyngeal cavity. Second section: mastication movements. A 3 kg common carp was tranquilized (but not anesthetized) to enable exploration of the pharyngeal cavity with conservation of mastication movement induced by pharyngeal mucosa stimulation with the endoscope. Click here for file
